# Determination of linear expansion of AlSi10Mg aluminium alloy depending on external conditions during solidification

**DOI:** 10.1016/j.heliyon.2022.e11363

**Published:** 2022-11-02

**Authors:** F. Radkovský, M. Gawronová, N. Válková, P. Lichý, I. Kroupová, V. Merta, I. Nguyenová

**Affiliations:** a Department of Metallurgical Technologies, VSB-Technical University of Ostrava, Faculty of Materials Science and Technology, 17. listopadu 2172/15, 708 00, Ostrava, Czech Republic; b Brembo Czech, s. r. o., Na Rovince 875, 720 00 Ostrava, Czech Republic

**Keywords:** Gravity casting, Silumin, Metal mould, Thermal expansion, Hardness, Microstructure

## Abstract

The article deals with the possibility of influencing the properties of aluminium alloy castings by changing the external conditions during solidification. Due to the high demands of customers, influencing the resulting microstructure is an important process in the production of castings. When casting an aluminium alloy into a metal mould, the mould must be preheated to a high temperature. For this reason, the key parameter of production is the mastery of the control of the resulting microstructure of castings and the related internal quality and mechanical properties, which are subject to high demands defined by international standards.

The aim of the experiment is to evaluate the influence of different preheating of the metal mould on the resulting structure of test castings made of AlSi10Mg material and its subsequent thermal expansion. It is hypothesized that as the temperature of the mould preheating increases, the mechanical properties will deteriorate and the expansion of the alloy will increase. In the case of immediate use of castings after production, this could have a negative impact on the service life and safety of entire assembled components. All measured results will be put into comparison and direct connections between the data will be searched using the microsoft excel program.

## Highlights

• Comparison of different preheating of permanent moulds.
• Use of Alsi10Mg alloy as a representative of the most widely used silumines.
• Comparison of mechanical and physical properties.
• Finding connections between measured values.
• Influence on thermal dilatation.

## 1 Introduction

Foundry silumines, i.e. alloys of aluminium with silicon, have many advantages in terms of a combination of good technological and mechanical properties. They excel in excellent foundry properties (good running quality, narrow solidification interval), as well as corrosion resistance, low density in combination with sufficient strength. These are “universal” materials useable for various technologies and types of resulting castings. Due to possible applications of Al–Si alloys involving high mechanical and thermal loads (e.g. pistons, cylinder heads, brake straps and engine bodies), an alloy with high corrosion resistance, good mechanical properties and dimensional stability at different working temperatures is required [1, 2]. For the engine components of cars and other vehicles, a material is usually chosen that is designed for heavy-duty use, especially the aforementioned aluminium-silicon alloy for foundry purposes. The fatigue properties of this material can be described by specific microstructure characteristics. The microstructure parameters are always influenced by the cooling effect of the mould during casting, with even greater emphasis on castings that have variable wall thicknesses or a high metal volume. In the structure, we observe the porosity values, the morphology of the eutectic silicon and the spacing of the secondary dendritic arms (SDAS) [3, 4, 5, 6, 7, 8, 9, 10, 11, 12].

The AlSi10Mg alloy is one of the hypoeutectic silumines whose structure consists of a network of primary dendrites of a solid solution of α(Al) and an eutectic separated in interdendritic spaces [13, 14]. There are a number of parameters that affect the resulting microstructure – melt purity in terms of inclusions and dissolved gases, chemical composition (use of alloying elements), heat treatment of already finished castings or solidification conditions or cooling of castings (especially the rate of cooling). The cooling rate is discussed by the authors in articles [15, 16], where they just evaluate structural changes in castings. Under common operating conditions, permanent metal moulds are most often used for casting the Al–Si alloys, which generally show a good cooling effect, thanks to which the resulting fine-grained structure of castings is also ensured. However, these metal moulds must be preheated to a high working temperature due to the increase of the melt running quality. Increasing the running quality of aluminium alloys is discussed by the authors in the article [17].

Silumines tend to solidify by volume and crystallization of eutectic occurs at a temperature lower than the eutectic temperature (α + Si), therefore the eutectic is locally isolated from the filling with liquid metal. This affects both macroscopic and microscopic porosity in the casting structure. The hydrogen contained in the liquid metal forms a gas porosity and it is therefore usually present in castings [18].

In the case of a part with a high accuracy requirement and at the same time a low dimensional tolerance, permanent dimensional stability is one of the basic requirements [19]. This characteristic depends on the final structure of the aluminium castings. It is known that silumines form metastable structures after casting, even more conspicuous after heat treatment carried out to improve mechanical properties. The presence of alloying elements in industrial silumines, which generally improve mechanical properties, at the same time affect their coefficient of thermal expansion. The authors [15, 20, 21] are studying castings with thermal stress for components of car driving mechanisms, where they focus, for example, on the assessment of mechanical properties at high temperature and achieve improvements by adding Mn or Ni. The authors [22] describe that solidification rate, zinc and magnesium concentrations as well as silicon and iron concentrations affect the amount, scale and constitution of eutectic structures and porosity. The process of deterioration of the coefficient of thermal expansion can be avoided by additional heat treatment for dimensional stabilization. If this is not possible, the maximum operating temperature is limited under the temperature where the metastable state transforms [23].

As already mentioned, the microstructure of the material can be influenced by the use of alloying materials, but also by the refining of the melt or subsequent heat treatment. When describing the microstructure of silumine, such microstructural parameters as, for example, the spacing of secondary/dendritic arms (SDAS), the shape and size of eutectic silicon crystals (PSPA), the proportion and dimensions of intermetallic phases, and porosity are evaluated [1, 24, 25].

. As stated by the authors [26], SDAS is an important parameter for the designer of a cast part. If we measure this parameter in different parts of the casting we obtain values that are influenced by the heat dissipation rate. Higher cooling rate will reduce the solidification time, and will also reduce the SDAS parameter which will improve the mechanical properties of the aluminium alloy. This statement is acknowledged by other authors [27, 28, 29, 30]. As study [26] describes that SDAS is approximately equivalent to the third root of the solidification time. Therefore, we can confidently conclude that the quality control of castings, with respect to the mechanical properties of the aluminium alloy, can be verified on microstructure images by measuring the SDAS parameter. The SDAS values in the observed problem areas are also important because they have an influence on the occurrence of other defects. The SDAS values can then also be specified in the customer’s requirements.

It is certainly necessary to investigate and evaluate the dependencies between cooling method, metallography, because for example, studies [12] have shown that material fatigue experiment and fatigue crack propagation on aluminium alloys used for mass production confirm the effects of secondary dendritic arm spacing (SDAS) on cooling rates. In this experiment, test samples were taken from two materials. The first was AlSi8Cu3 alloy from the engines and the second was AlSi7Cu0.5Mg alloy from the cylinder heads. Testing was carried out under uniaxial cyclic mechanical loading.

By influencing the metal solidification process, microstructural changes in the material can also be achieved, for example, the high cooling rate allows the eutectic silicon phase to be refined, which significantly affects the mechanical properties of the material, especially it has a positive effect on its fragility [25, 31].

A prerequisite would therefore be the choice of a mould with the highest cooling effect without preheating or the application of additional rapid cooling of the mould after casting. Under common operating conditions, permanent metal moulds are most often used for casting the Al–Si alloys. However, these metal moulds must be preheated to a high working temperature in order to increase the running quality, but in order to extend the service life of the moulds and increase the requirement for production efficiency, i.e. the number of casting cycle repetitions per hour, the moulds cannot be shock-cooled immediately after casting. The production of a permanent mould (one piece or a small batch) is very expensive and therefore has to manage to produce up to 100,000 quality castings before it is scrapped. Even the initial production must be precise and the mould must be useable immediately. The appropriate shape and parameters must be designed so that the desired metallurgical properties of the final products can be achieved. One of the main objectives is to reduce production costs, reduce scrap (castings discarded) and improve quality. This can be achieved by the correct mold parameters, where the designer must be knowledgeable in the areas of material physics, heat sharing and transfer, flow and solidification [26, 32, 33].

Therefore, it is also necessary to deal with the influence of preheating of metal moulds on crystallization and solidification and the resulting microstructure of cast parts.

The thermal stress of permanent moulds and then the structural and metallurgical properties of the cast alloy are affected by the mould preheating parameter. As shown in the study [34], it was found and confirmed that increasing the preheating temperature of the mould increased the solidification time of the castings (reducing the cooling effect of the mould). The observed samples then had a coarse-grained internal structure [35]. The influence of microstructure is also evident on the thermal dilatation of castings, and therefore, instead of the conventional method of monitoring solidification curves (TA), when cooling the casting, the thermal dilatation can be monitored and evaluated on already solidified samples [1]. The monitoring of thermal expansion or TA is dealt with by the authors in the articles [36, 37, 38]. They monitor here the influence of alloying elements (Si, Cu, Ni), coefficient of thermal expansion and also the resistance to thermal shocks. In the case of the addition of graphite, the decrease in thermal expansion was measured by dilatometry.

The coefficient of thermal expansion of Al is higher in order than Si, and therefore the thermal stresses are formed due to the thermal inconsonance between the Al phase and the Si particles, thus providing a driving force for recrystallization [39]. The dilatometric test measures the coefficient of thermal expansion of a sample over a wide temperature range. The coefficient of thermal expansion is an important property in many scientific branches for determining dimensional stability. During the rise or fall of temperature, the base phase in the alloy does not expand or shrink evenly. This can cause permanent deformation in the metal matrix. Currently, data on the behaviour of thermal expansion in Al–Si–Mg alloys rarely appear in the published literature [39].

The measurement of the study is to compare the solidification conditions of the AlSi10Mg alloy depending on the temperature of the mould preheating (25, 100, 200, 350 °C). The research will be focused on finding out how the defined temperatures of the mould preheating will affect: thermophysical and mechanical properties of the monitored alloy. In conclusion, a correlation is established between the results of solidification of test samples in metal moulds preheated to different temperatures, thermophysical properties and the micro and macro structure of the studied alloy. To verify the chemical composition of the metal, a spectral analysis will be performed.

The aim of the study is to determine the evolution of differences in the properties of the material cast into different preheated moulds with different cooling times. In automotive moulds are preheated to different temperatures and therefore it is important to verify the effect on the casting parameters. The data obtained will be helpful in optimizing the production process.

## 2 Materials and methods

### 2.1 Choice of the alloy

The alloy ALSi10Mg was chosen, which complies with the EN1706:2020 (ENAB43000) standard. Before the experiment the chemical composition was checked and, as can be seen in Table 1**,** the measured values correspond to the standardized composition. The AlSi10Mg alloy was selected due to a request from our industrial partner in the automotive sector, who is considering this material as an alternative to AlSi7Mg0.3.

**Table 1:** Chemical composition of the studied aluminium alloy in % by weight.

	Si [%]	Fe [%]	Cu [%]	Mn [%]	Mg [%]	Ni [%]	Zn [%]	Pb [%]	Sn [%]	Ti [%]
Average values	9.35	0.2	0.01	0.02	0.26	0.002	0.01	Not measured	0.002	0.1
Values according the standard	9–11	0.4	0.03	0.45	0.25–0.45	0.05	0.10	0.05	0.05	0.15

### 2.2 Manufacture and preparation of samples

**Figure 1:**
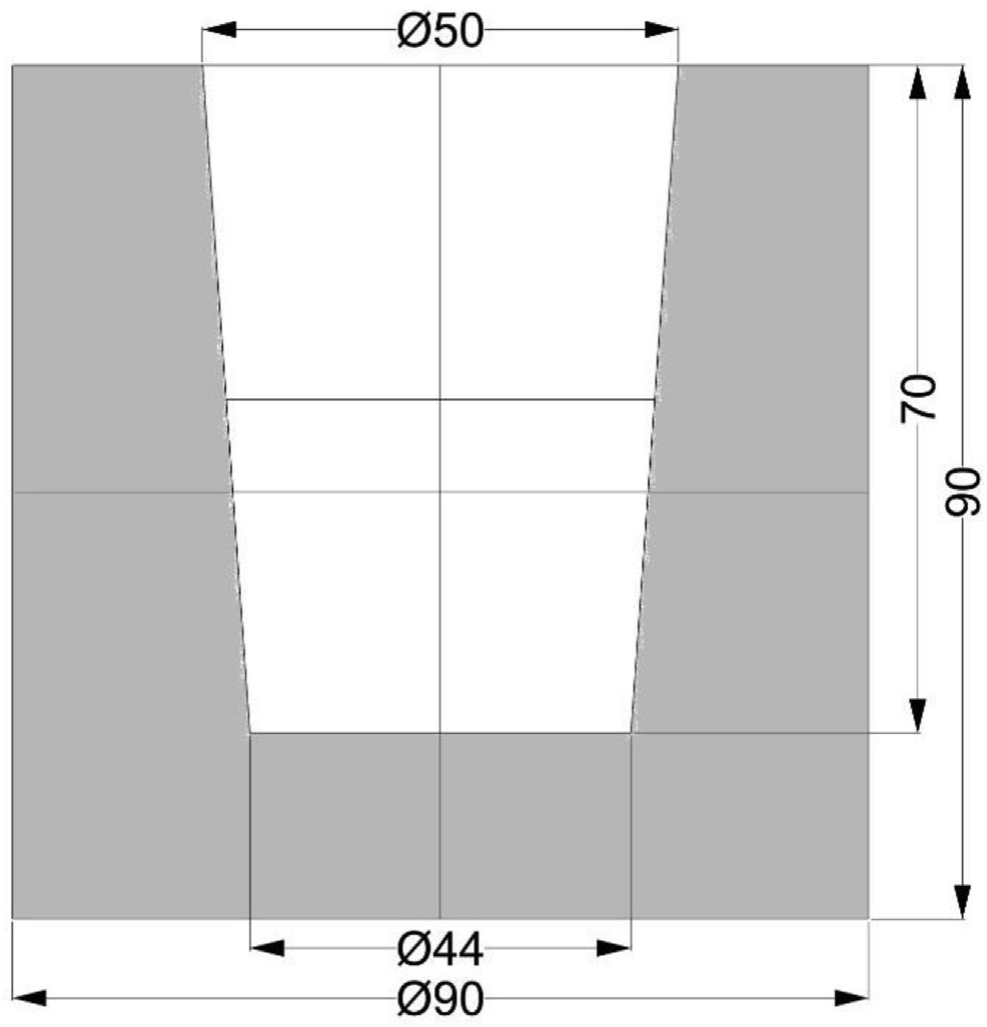
Drawing of the metal mould (accuracy: ISO 2768-m).

The temperature on the melting furnace was set at 740 °C and the melting process took 4h ± 5 min. The weight of the charge was about 1 kg. Casting took place at a melt temperature of 710 °C into a preheated steel mould. The height of the casting was 70 mm and the diameter was 50 mm. The geometry of the coccyx was chosen to achieve the most even heat dissipation. The designed size of the permanent mould allowed easy removal of the casting and obtaining a sufficient number of samples for evaluation. Figure 1 shows a drawing with the dimensions of the metal mould. The metal mould was made by machining from a 100 mm diameter bar of S355J2 quality. The metal mould used for casting the samples was made according to known regularities, and after pulling out of the furnace it was placed on the spikes to allow natural cooling even on the underside of the mould, as indicated in the article [40].

**Figure 2:**
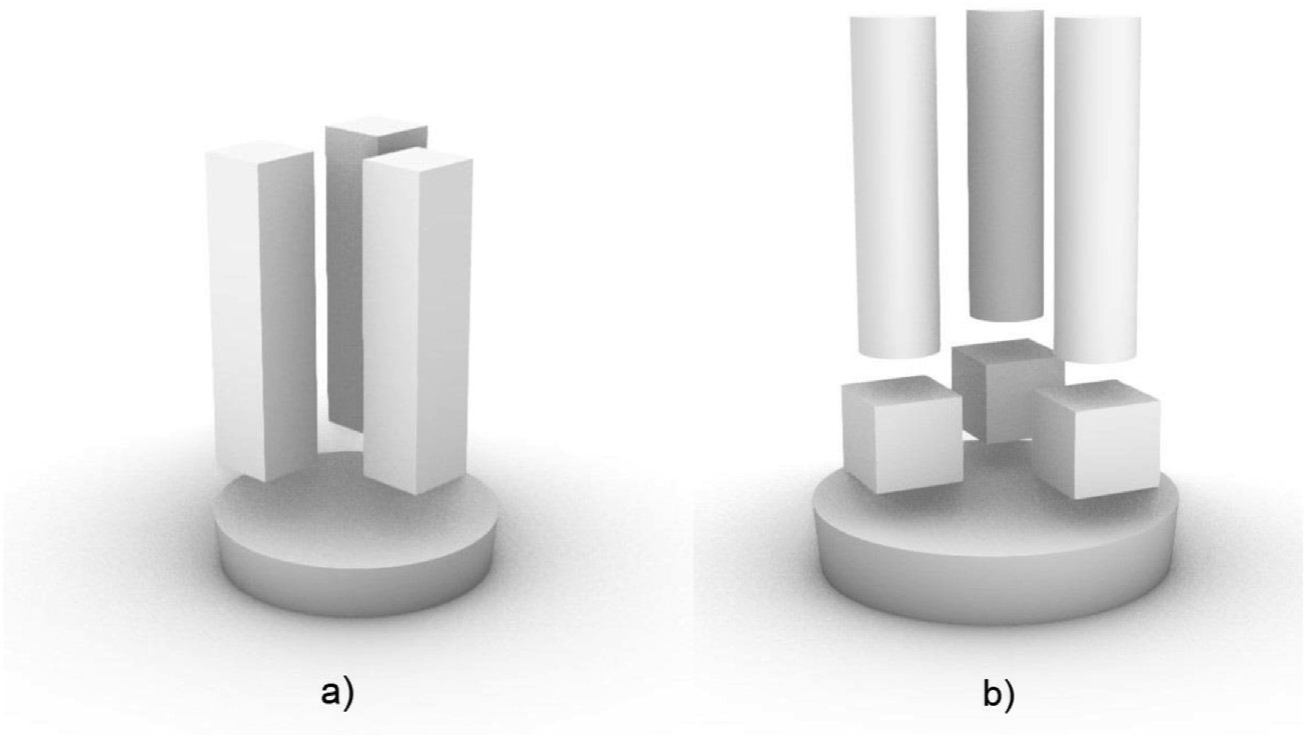
(a) Semi-finished product for dilatometry, samples for metallography and microhardness, (b) sample for spectral analysis, three samples for metallography, microhardness of the size 10 × 10 × 10 mm and three rollers for dilatometrical tests with a length of 25 mm and a diameter of 6 mm.

The metal mould was preheated in a side furnace to the required temperature (25 °C, 100 °C, 200 °C, 350 °C). When we chose the preheating temperatures, we based them on real-life practice and the requirements of our industrial partner. Preheat temperatures of 25 °C–350 °C were chosen as alternative preheat temperatures to monitor changes in material properties. The casting itself took place just in this furnace in order to keep the mould temperature at the desired temperature. After casting, the moulds were pulled out of the furnace and allowed to cool in air to a temperature of 300 °C ± 10 °C. After reaching the mentioned temperature of 300 °C on the surface of the casting in the open part, the casting was tilted out and left to cool freely in the air. The temperature on the casting surface was measured using a thermocouple. Two identical samples was always the result from each melt. Further on the samples were divided into smaller parts for individual tests (Figure 2a, b), i.e. test set. The cut off bottom part was further used for spectral analysis. From three small prisms cut from each sample, small cubes were separated for metallographic and mechanical tests. These cubes were separated from the prisms from the side closer to the bottom of the mould. From the rest of the prisms the pieces were made for the test of linear thermal expansion by machining. Table 2 provides data on the samples, namely the markings and their number. A verification set of samples was made in the same way to verify the individual measurements.

**Table 2:** Marking of samples.

Marking of samples	Casting temperature [°C]	Temperature of the metal mould [°C]	Number of base samples	Total number of studied samples for the given temperature
25	710	25	2	14
100	710	100	2	14
200	710	200	2	14
350	710	350	2	14

### 2.3 Evaluated properties

The alloy was melted in an electric resistance chamber furnace LAC with a box door in a carbide crucible. Metal moulds were heated again in an electric resistance chamber furnace LAC with an inner chamber size of 50 × 50 × 50 cm.

Spectral analysis was performed on the Bruker Q4 TASMAN device. This is a spark analyzer. Before the measurement, the calibration was always carried out using the corresponding standards.

Metallographic samples were carried out on a semi-automatic device Labopol-5 of the Struers mark. Abrasive papers with standardized grit and also standardized order were used for grinding.

The Axio Observer A1 microscope from Zeiss connected to the Promica camera of the PROMICAM 3-3CP mark was used to take pictures of the microstructure. Subsequently, the computer software QuickPHOTO INDUSTRIAL 3.1 was used to focus and store the photos. Microhardness and the SDAS parameter were evaluated in the pictures.

The SDAS parameter can be evaluated for example manually (this method is used by the authors in articles [41, 42] or automatically (the authors in the experiment in the article [43]. Using the evaluation of the SDAS parameter the authors of the article [44] also attempted and successfully verified the influence on the corrosion of the aluminium alloy. There have been attempts to predict the SDAS parameter, such as the authors in the article [1]. In our case, the measurement was carried out manually as a standard. To identify and measure groups of secondary axes of dendrites using pattern analysis (also known as PA), it was necessary to set the threshold of each photograph and mark the measured dendrites. The value of the distance of the dendrites axes was automatically determined according to the relation (1):

(1) S*DAS = L/n*.*M*,

where *L* is the length of the line segment, *M* is the used magnification, and *n* is the number of secondary dendrites intersected by the measuring line.

The DuraScan 70 G5 Brinell hardness tester from Emcotest was used to measure hardness. It is a fully automatic hardness tester that is controlled using the ecos Workflow CIS Pro computer software. The method of performing this test is described by the ČSN EN ISO 6506-1 standard. The test is denoted by the abbreviation HB.

Finally, a dilatometer from Netzch with the designation DIL 402/C was used to measure linear thermal expansion. The apparatus was equipped with corundum components (tube, carrier, rod, pads). Before the individual measurements, the apparatus was corrected by a corundum correction sample. Linear thermal expansion was measured on samples with a diameter of 6 mm with a length of 25 mm (±0.01 mm). Individual samples of the alloy were inserted into the apparatus with a static inert atmosphere (argon 6.0).

## 3 Results

From the test set, a total of 14 samples were made for each preheating temperature of the permanent mould, on which individual tests were carried out. A verification set of the same number of samples was made and verified in the same way. The differences between the results of the verification and test sets were less than 5% and are therefore not considered statistically significant. For this reason, the results of the verification set were not further included in the presentation of the measured values.

### 3.1 Microstructures

**Figure 3:**
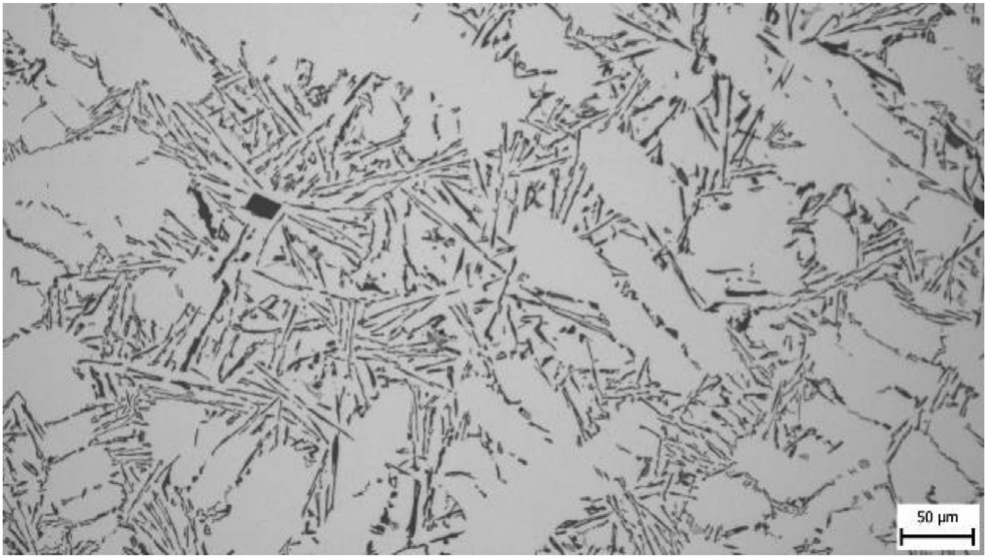
Sample 200.

From the taken pictures Figure 3 is given as an example. At a greater magnification (200 x), it can be seen that the silumine microstructure is formed by a matrix (a solid solution of aluminium) in the form of dendrites and an eutectic formed by silicon and intermetallic particles. The needle structure of eutectic silicon is visible in the figure.

**Figure 4:**
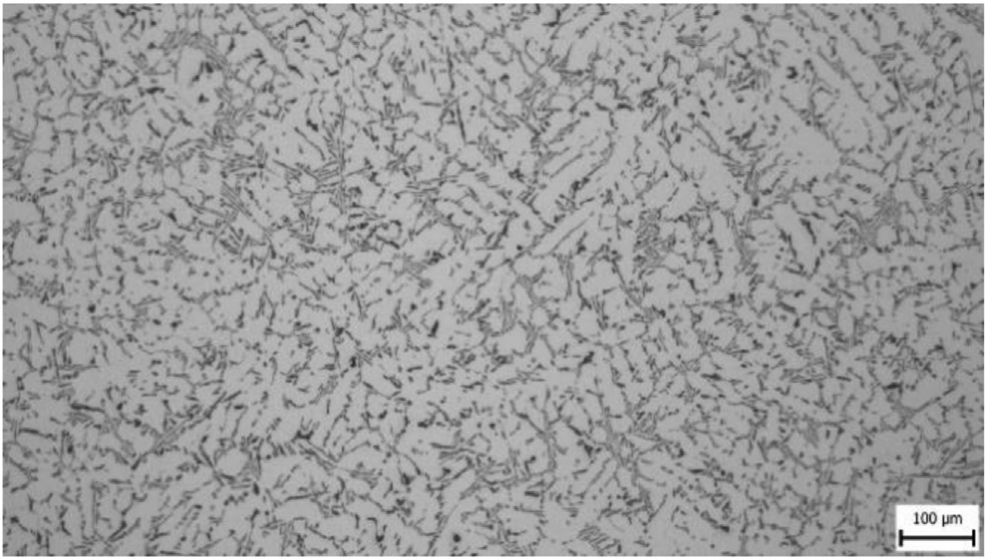
Microstructure of AlSi10Mg in the as cast state – 25.

**Figure 5:**
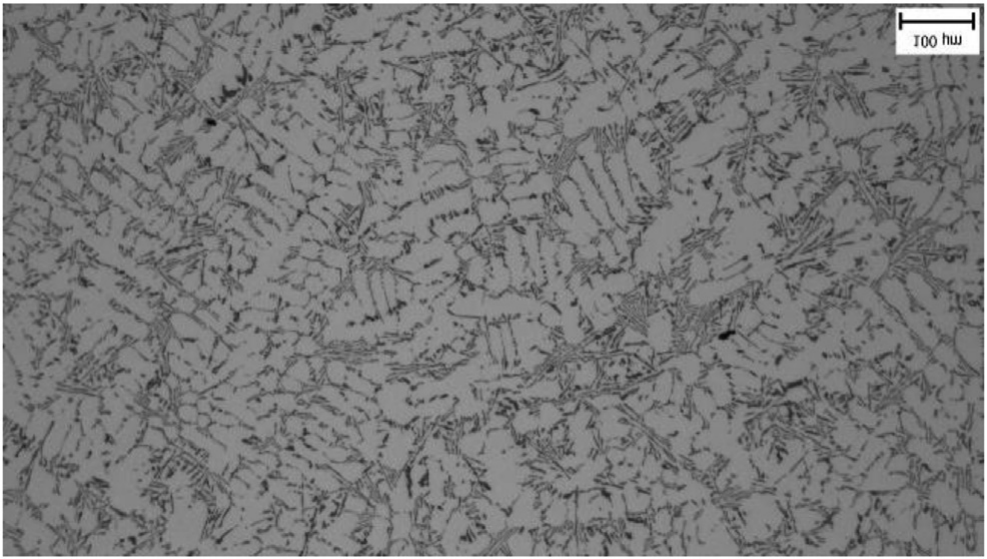
Microstructure of AlSi10Mg in the as cast state – 100.

**Figure 6:**
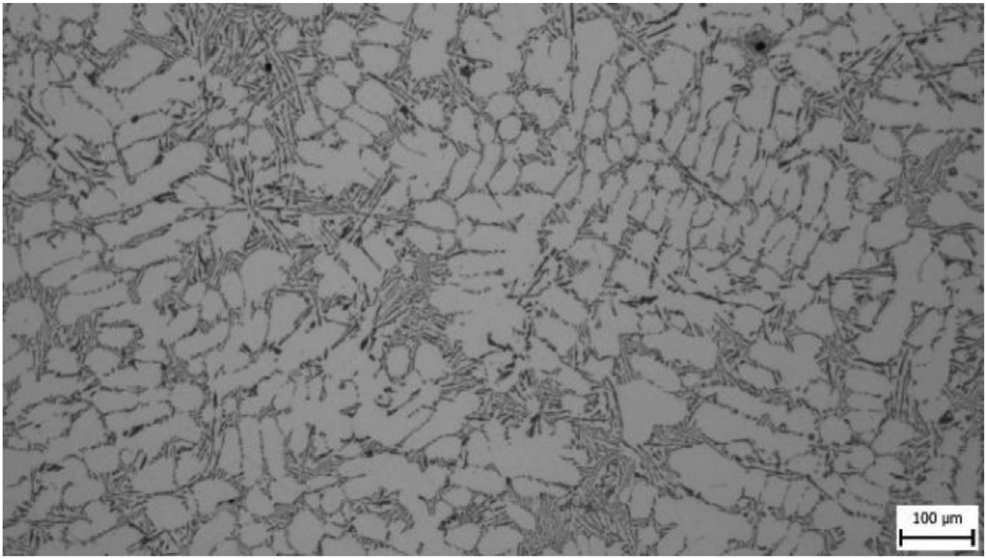
Microstructure of AlSi10Mg in the as cast state – 200.

**Figure 7:**
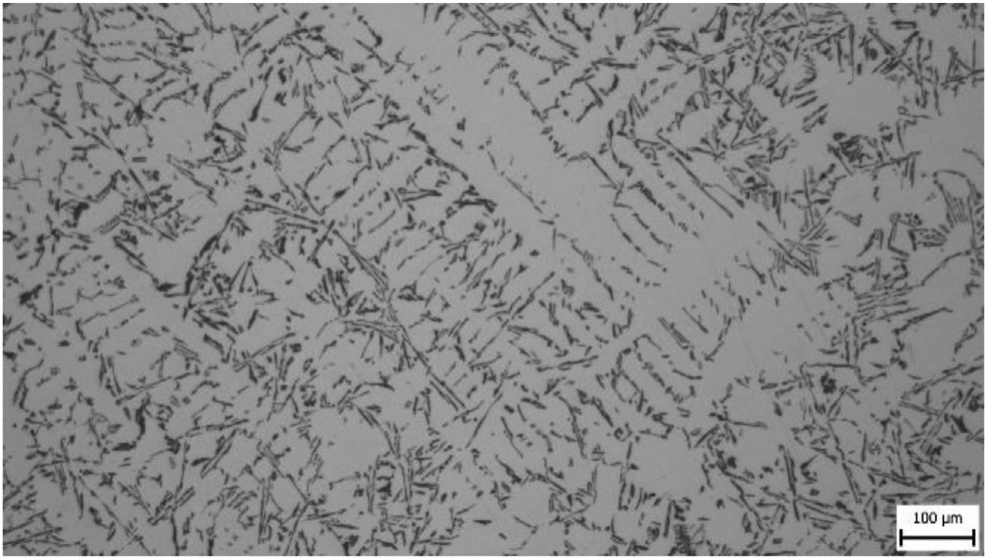
Microstructure of AlSi10Mg in the as cast state – 350.

Microstructures were evaluated for all samples and it was assumed that differences in microstructural parameters would be present. Figures 4–7 show photographs of microstructures of samples. The microstructures provided a sufficient set of results for evaluation.

### 3.2 Amount of separated eutectic

From the taken pictures (at a magnification of 100×), the percentage of separated eutectic was further evaluated. The eutectic was evaluated with the QuickPHOTO INDUSTRIAL 3.1 program. The values for each sample have been averaged and entered in Table 3.

**Table 3:** Summary measurement results.

Sample/temperature of the mould [°C]	25	100	200	350
Average proportion of the eutectic [%]	19.09	18.11	17.99	17.75
Standard deviation	1.10	1.17	0.86	1.32
Average hardness	67.25	64.85	64.75	62.60
Standard deviation	2.80	2.70	3.35	2.30
Average microporosity [%]	0.04	0.04	0.04	0.02
Standard deviation	0.08	0.05	0.04	0.03
Average SDAS [μm]	27.21	26.64	26.71	29.44
Standard deviation	1.01	0.57	1.63	1.05

### 3.3 Measurement of the Brinell hardness

To measure the hardness of silumine, the Brinell method of measuring the hardness of metals was used [45]. The achieved average values are shown in Table 3. The resulting values ranged from 67 to 62.

### 3.4 Evaluation of microporosity

The microporosity was evaluated to control the internal quality using the optical microscopy. On the taken photos (at a magnification of 100×) it was possible to carry out the analysis. Of the measured values the porosity ranged from 0.021 to 0.039%. All averaged values are recorded in Table 3. According to the results it is evident that the samples were almost homogeneous.

### 3.5 Evaluation of the SDAS parameter

The evaluation was carried out manually and about 500 secondary axes of dendrites were measured for each sample. The resulting values ranged from 26 to 30 μm. Average measurement results are shown in Summary Table 3.

### 3.6 Dilatometric measurements (thermal expansion)

From each defined temperature (25, 100, 200, 350) 6 samples were prepared. The temperature increase was set to 10 K/min with a temperature increase of 25–530 °C.

**Figure 8:**
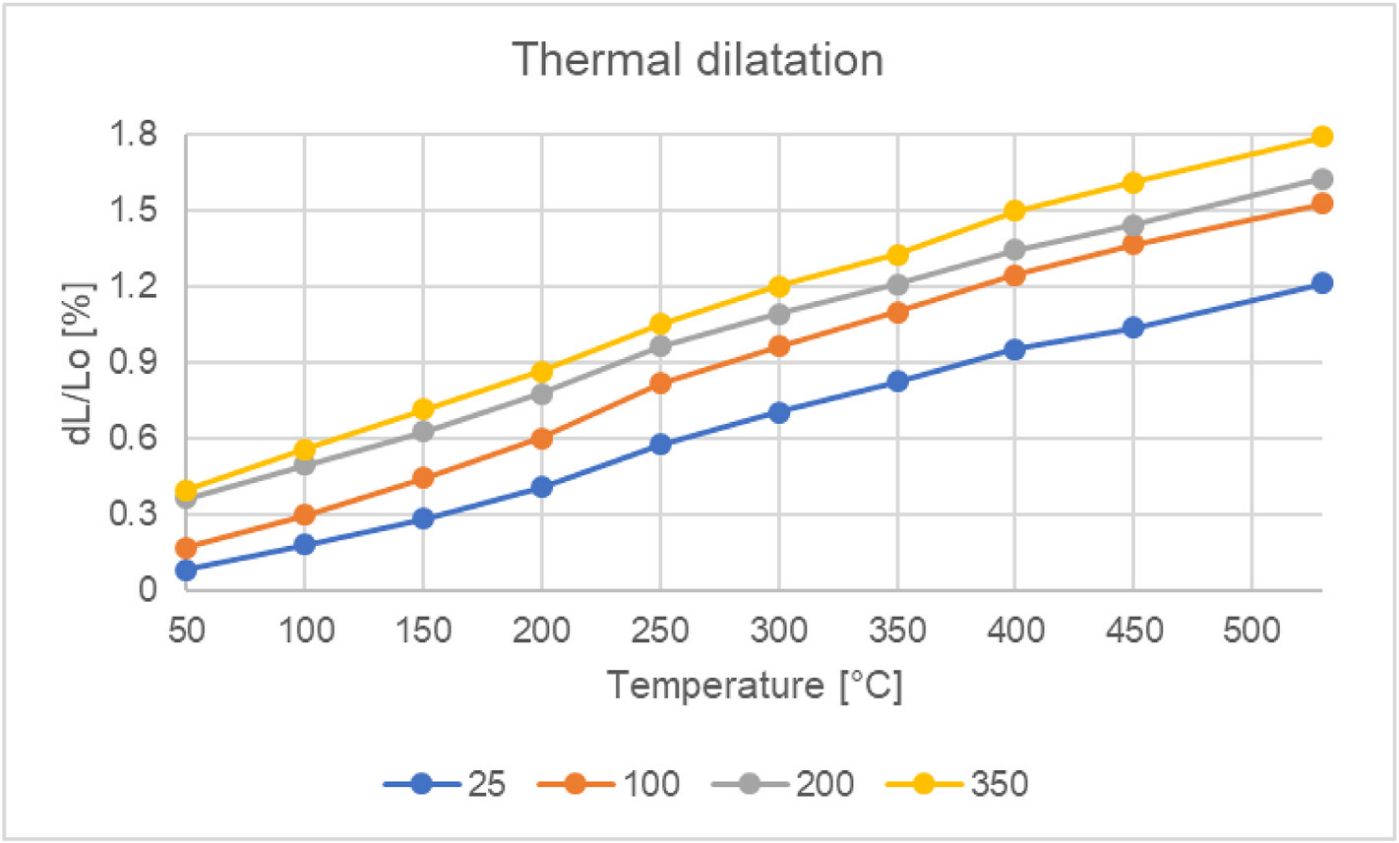
Graph – dependence of thermal dilatation of AlSi10Mg alloy on temperature.

Figure 8 shows the dependence of the average value of the measurement of thermal dilatation on the heating temperature of the sample.

The values of the samples cast into the metal mould (25 °C) achieved an average thermal expansion dL/Lo_Ref._ = 1.214% (a reference value). At mould temperatures of 25, 100, 200 and 350 °C, the average dilatation was dL/Lo_25_ = 1.214; dL/Lo_100_ = 1.527; dL/Lo_200_ = 1.627; dL/Lo_350_ = 1.792%. The highest increase of dilatation of all the given results was therefore evident in samples cast in the 350 °C mould, where the difference between dL/Lo_Ref._ and dL/Lo_350_ was 0.578%. The second highest difference was recorded between dL/Lo_Ref._ and dL/Lo_200_, and namely 0.413%. In the last comparison between the reference value and samples cast in the 100 °C mould, the difference was the lowest, dL/Lo_Ref._ and dL/Lo_100_ = 0,313%.

## 4 Discussion of results

An experiment was done to determine how the defined temperatures of the mould preheating would affect: structural, mechanical and thermophysical properties of the studied alloy. The obtained results of linear thermal expansion were related to microstructure, macrostructure [46], microporosity, amount of the separated eutectic, dendrite size (SDAS) and hardness (HB). The macroanalysis did not provide results that could be evaluated. The samples were clean with no pores. In case of microporosity, internal quality was checked that confirmed the internal homogeneity.

### 4.1 Proportion of the eutectic content and the value of the SDAS parameter

**Figure 9:**
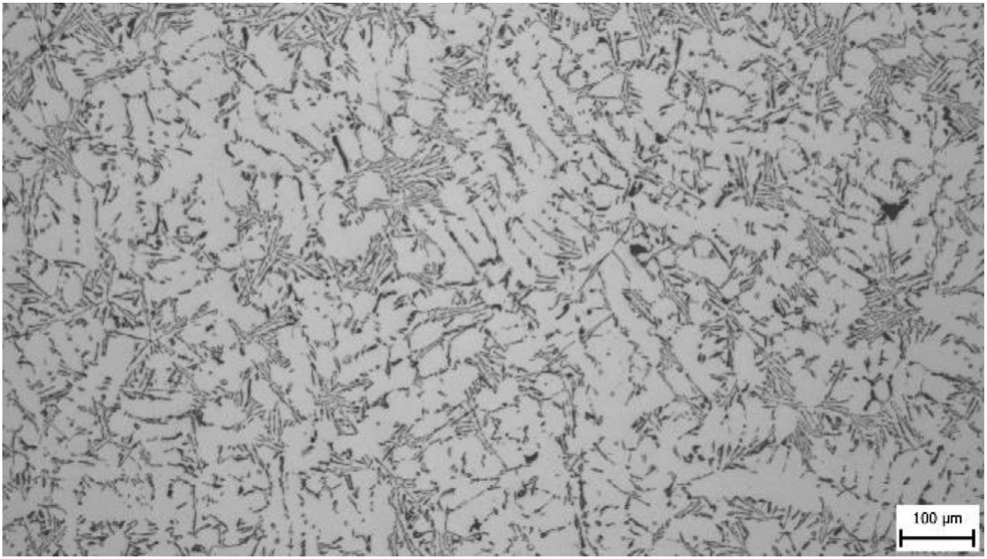
Microstructure of AlSi10Mg in the as cast state – 25.

**Figure 10:**
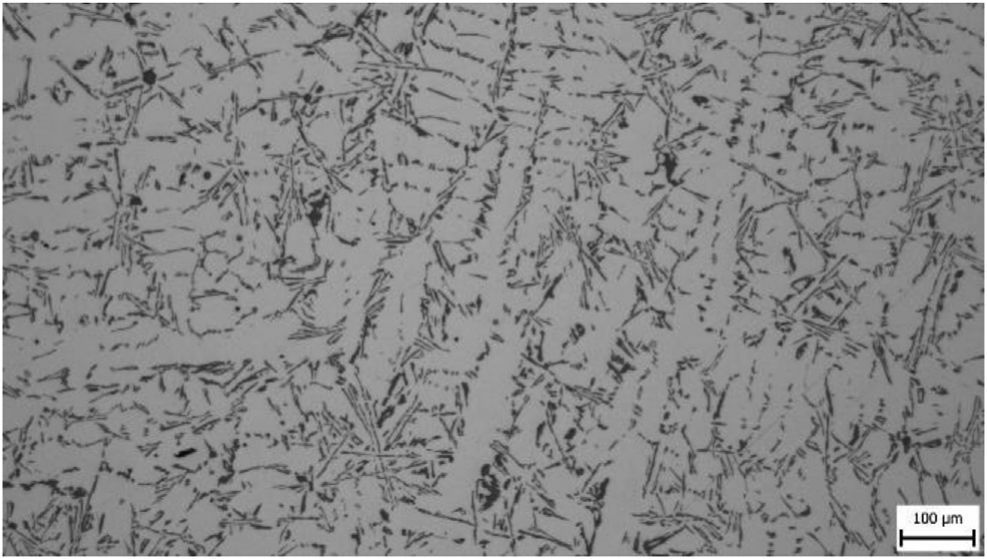
Microstructure of AlSi10Mg in the as cast state – 350.

**Figure 11:**
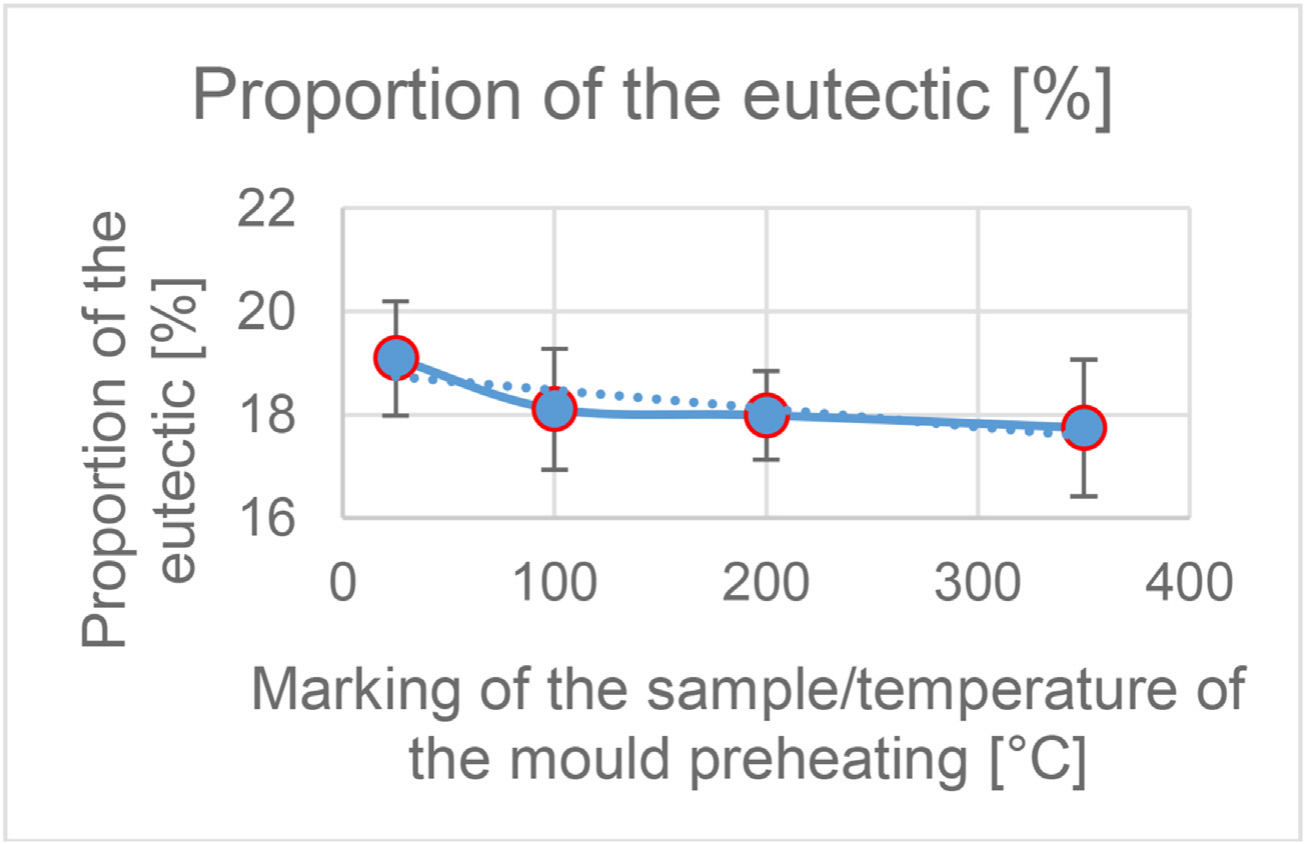
Graph of the dependence of the proportion of eutectic on the temperature of the mould.

Pictures of samples (Figure 10.) from a higher mould temperature show a smaller proportion of the eutectic than pictures at a lower temperature (Figure 9.). Figure 11 shows a graphical representation of the influence of the content of the separated eutectic at the preheating temperature of the permanent mould. The proportion of the eutectic content decreases with increasing temperature of the mould. The difference in the results of samples 25 and 350 was up to 7%.

**Figure 12:**
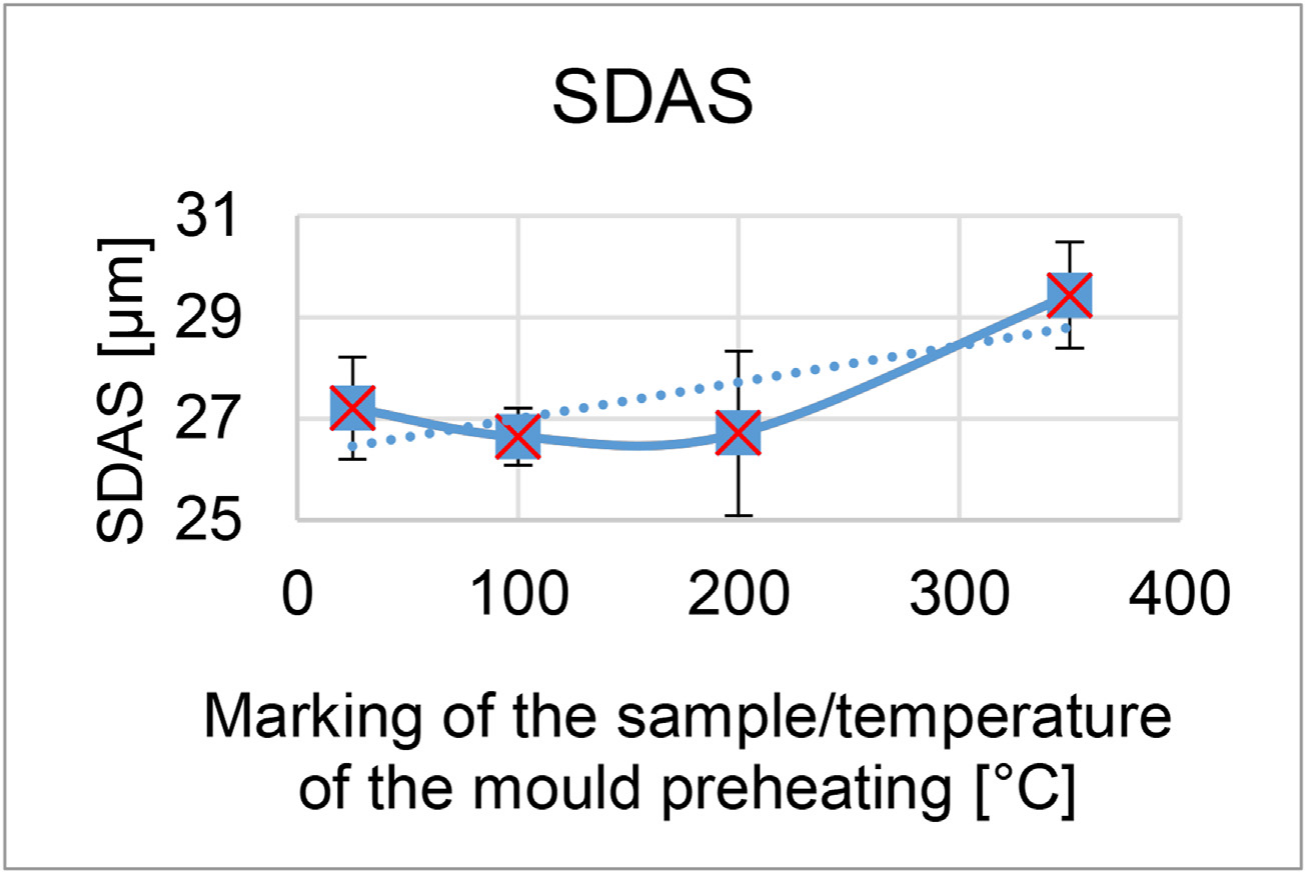
Graph with SDAS values for individual samples.

The results show the influence of the different temperature of the mould heating on the microstructural parameter of SDAS. For clarity, the values are given in a separate graph (Figure 12.)

In microstructure pictures (Figures 4, 5, 6, and 7) where the SDAS parameter was evaluated, several dendritic arms are still clearly visible during casting at the mould temperature above 200 °C. The difference is in the shape of secondary or tertiary branches. At a lower temperature, these branches are smaller and narrower. This trend continues until the room temperature, when the cooling of the melt is high and the change of dendrites is also evident from the structure. They are smaller and rather round instead of the oblong shape. In this case the influence of mould preheating on the resulting structure has been confirmed. The microstructure images of the extreme preheating values (Figures 9 and 10) show a significant thickening of the structure when the coccyx is preheated to 350 °C compared to preheating to 25 °C. This is confirmed by the SDAS result, where the 350 sample achieved 2.23 μm, or 8.2%, larger average dendrite size than the 25 sample.

**Figure 13:**
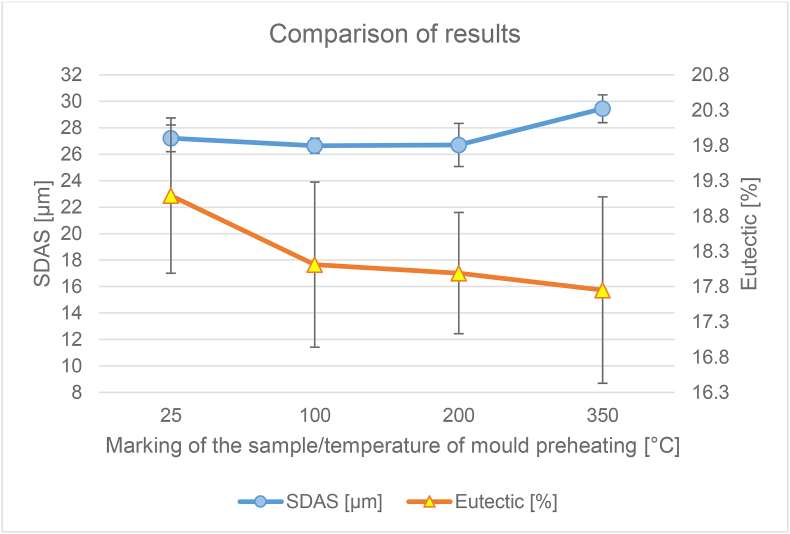
Graph of the dependence of the proportion of eutectic and SDAS parameter on mould temperature.

The dependence of these two parameters (the content of the separated eutectic and SDAS) on temperature is recorded in Figure 13. After inserting the trend line into the graph for both studied parameters, an upward trend can be seen in the SDAS value and in the eutectic the trend on the contrary decreases. Values of the eutectic proportion ranged from 17.75 to 19.09% of the eutectic in the alloy. It can be noted a slight decrease of the eutectic proportion with increasing temperature. For SDAS values the values ranged from 27.21 to 29.44 μm.

From the reference temperature of the metal mould of 25 °C there was a slight decrease of the parameter and at a temperature of 200 °C there was an increase. The difference between the minimum and maximum values of the parameter was up to 7.5%. When the permanent mould is preheated to 100 °C and 200 °C, it can be said that the eutectic fraction and the SDAS parameter do not change significantly. Therefore, from the measured results it is clear that the selection of these operating temperatures will not have a negative effect on the observed properties of the alloy and can be chosen according to the needs of the specific casting.

### 4.2 Hardness and the SDAS parameter

**Figure 14:**
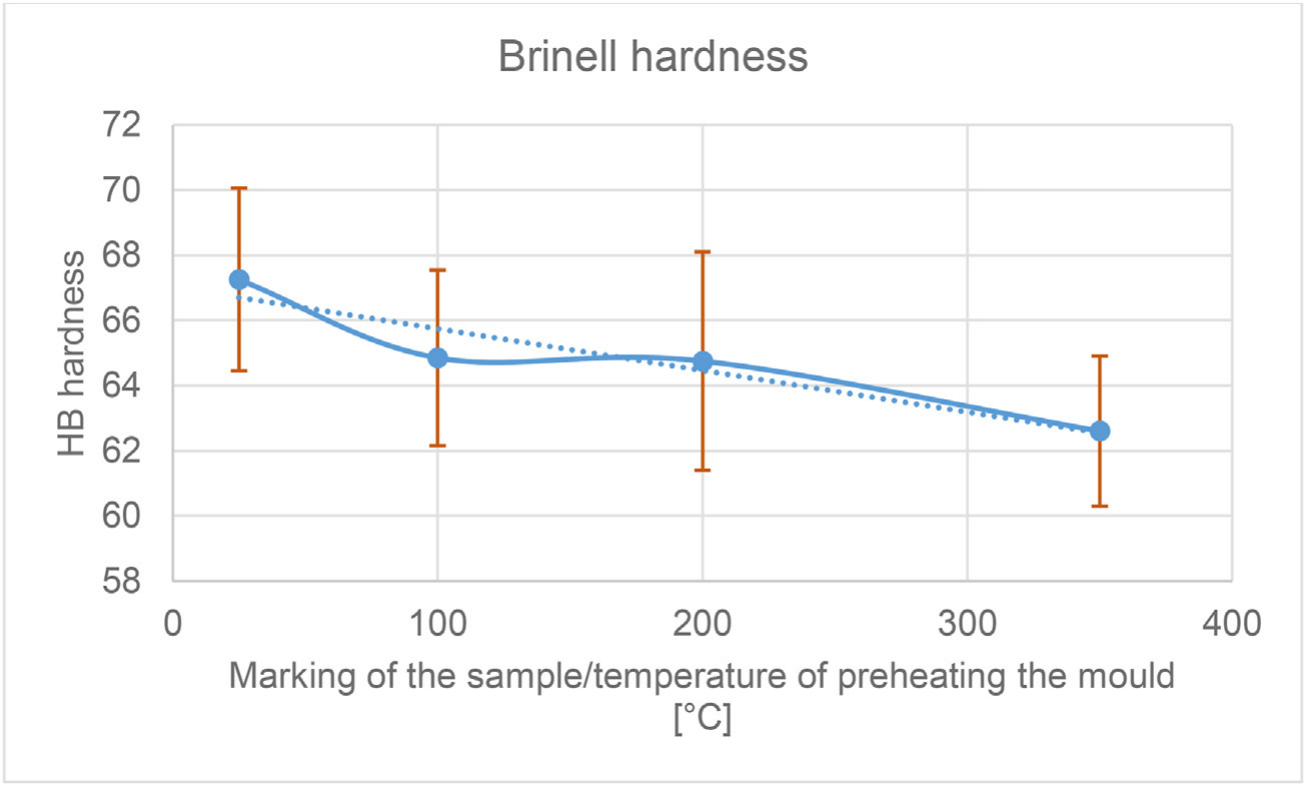
Graph of the dependence of the Brinell hardness value on the mould temperature.

**Figure 15:**
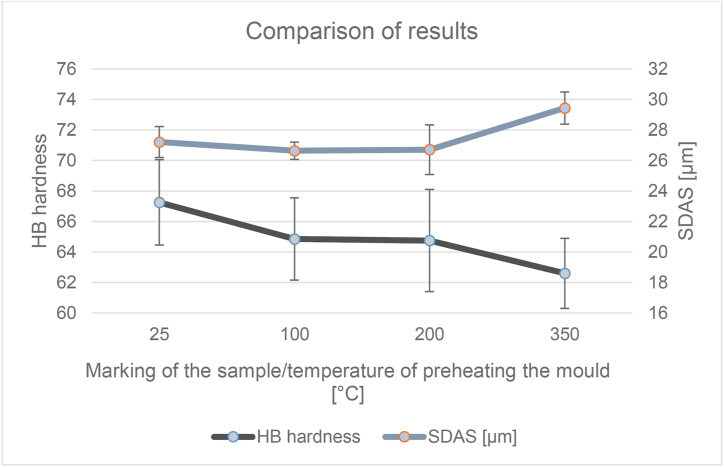
Graph – Comparison of hardness and SDAS results.

Hardness was an interesting parameter for evaluating the influence of preheating the metal mould. The differences in the achieved values range from 62.6 to 67.25. Figure 14 shows the dependence of the hardness value on the preheating temperature of the permanent mould. According to the prediction the hardness would to decrease with the increasing temperature of preheating the mould, and the results also confirmed this. Long dendritic arms just cause lower strength of the material. The range between sample hardness values of 25 and 350 is up to 7%. Figure 15 shows graphically a combination of decreasing hardness and a sharply increasing SDAS parameter.

Comparing the hardness results (Figure 14) for samples 100 and 200, we can also say that the hardness is comparable and thus no negative effect was observed. The alternative preheating temperature (sample 350) already shows us a certain negative influence for both lower hardness and higher SDAS (Figure 15). For sample 25 the measured hardness is the highest, the eutectic fraction is also the highest and the SDAS slightly increased. When the standard deviation is included, the increase in SDAS is statistically insignificant. Thus, a permanent mold temperature of 25 °C would provide the highest quality casting, but it is certainly not recommended because it would cause rapid heat dissipation and failure of the liquid metal to swell into the complex shapes of the metal molds. A permanent mould temperature of 350 °C has undoubtedly the worst results and it has been confirmed that it is not possible to increase the heating of metal moulds by leaps and bounds without adversely affecting the properties of the castings.

### 4.3 Linear thermal dilatation

The last and also the most important test performed was the thermal dilatation test. Articles by the authors [24, 31, 47] also deal with measuring the thermal dilatation. The authors emphasize the importance of dilatometric analysis, which is important for the evaluation of dimensional accuracy and stability under elevated temperatures. Silumines with the addition of Mg, Ni and Cu were also tested. There was an effort to positively influence – reduce – linear thermal expansion. In our case we did not change the chemical composition and the starting alloy was evaluated.

It was an assumption that the lower the cooling effect, the higher would be the thermal expansion values. This predication has been experimentally confirmed. The value of the thermal dilatation of a sample that was cast under room temperature is taken as a reference value. The values of these samples cast into a metal mould under 25 °C achieved an average thermal expansion of dL/Lo = 1,214%. The highest increase of dilatation of all the above mentioned results was evident in samples cast in the 350 °C metal mould dL/Lo = 1,792%, when the difference between dL/Lo_Ref._ and dL/Lo_350_ was 0.578% (Figure 12.). As mentioned above, internal porosity was not detected and internal homogeneity was confirmed in all samples. Therefore, we can exclude the claim that the higher porosity compensates for the expansion. When the linear expansion results are analysed, it is statistically confirmed that the highest expansion is in the samples with the highest SDAS parameter. At the same time, with higher eutectic content and higher nominal hardness, the linear expansion is the lowest.

**Figure 16:**
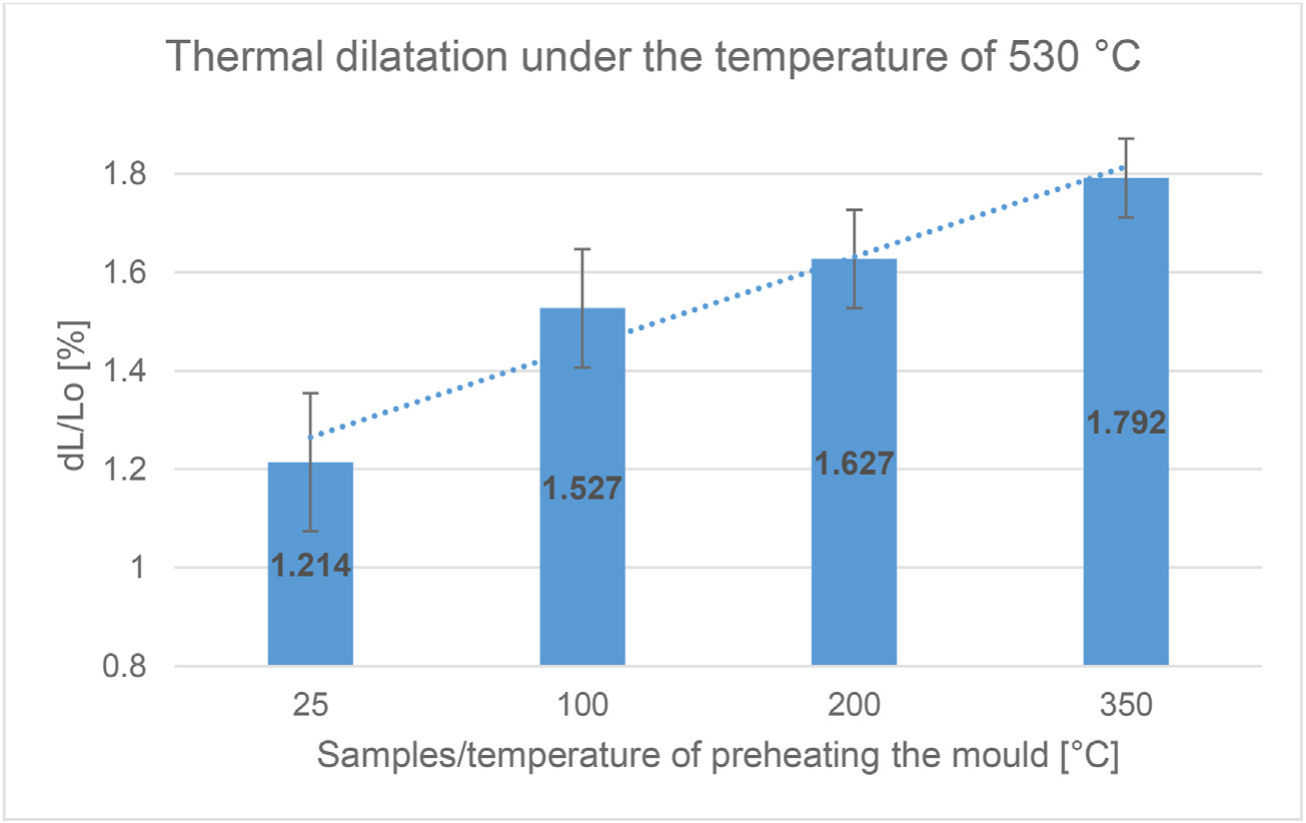
Graph – thermal dilatation of samples under the temperature of 530 °C.

The results of the final thermal expansion at the maximum measured temperature of 530 °C can be seen in the graph on Figure 16. We can note the confirmation of the hypothesis of the increasing form of the continuous and final value of the dilatation. This can be explained by the coarsening of the structure, the increase in the size of the dendrites accompanied by a decrease of hardness and a slight decrease of the content of the separated eutectic. There is therefore a noticeable difference between the preheating of the metal mould to a temperature of 25 and 350 °C, where the increase of dilatation is up to 32%.

## 5 Conclusion

In this work, the influence of different temperature of heating the mould before casting the AlSi10Mg alloy on its resulting properties was evaluated. The alternative preheating temperatures of 25 °C and 350 °C were chosen appropriately, resulting in the ability to monitor changes in the measured properties. The demonstrable influence has been confirmed and the conclusions are as follows.

1 – When evaluating the proportion of the separated eutectic, a downward trend can be noted according to the achieved values. Hardness was an important parameter for evaluating the influence of preheating the metal mould, and the evaluation itself yielded conclusive results that would confirm the negative influence on the mechanical properties of the studied alloy. It can be said with a support that there is a decrease of the hardness of the alloy with the preheating of the metal mould. The SDAS parameter was evaluated manually. The sequence of values showed a slight decrease and then a sharp increase. However, the results show the influence of the different temperature of heating the metal mould on the microstructural SDAS parameter. The upward trend with increasing heating of the metal mould from the heating temperature of 200 °C has been confirmed. When the permanent mould was heated to 100 °C and 200 °C, no demonstrable differences were found in the samples and therefore the choice of these temperatures is not confirmed to have a negative effect on the properties of the cast alloy. Both these preheating temperatures can be recommended for the use of cast alloy.
2 – Dilatometric measurements of linear thermal expansion yielded ascending results, where the value of dilatation after rounding ranged from 1.2 to 1.8%. The elongation of the sample increased with the increasing temperature of preheating the metal mould. The above mentioned results confirmed the unequivocal influence of the step-by-step increase of the mould temperature on the gradual increase of thermal dilatation of the test samples. For the samples with 100 °C and 200 °C permanent mould preheating, a difference of 0.1% was found in the resulting dilatation, and therefore both these temperatures can be recommended for the use of the cast alloy.
3 – It is recommended to continue in dealing with this topic and to include in the experiment also higher heating temperatures of the metal mould and subsequently also to subject to the research other used aluminium alloys. Specific alloys that are described as having increased heat resistance and dimensional accuracy at elevated temperatures could also be evaluated.

## Declarations

### Author contribution statement

Radkovský, F, Gawronová, M: Performed the experiments; Wrote the paper.

Válková, N: Performed the experiments.

Lichý, P: Conceived and designed the experiments.

Kroupová, I, Merta, V: Analyzed and interpreted the data.

Nguyenová, I: Contributed reagents, materials, analysis tools or data.

### Funding statement

This work was supported by 10.13039/501100001823Ministerstvo Školství, Mládeže a Tělovýchovy (CZ.02.1.01/0.0/0.0/17_049/0008399, Student Grant Competition SP2022/15, SP2022/68, Student Grant Competition SP2022/83, SP2022/84).

### Data availability statement

Data included in article/supplementary material/referenced in article.

### Declaration of interest's statement

The authors declare no conflict of interest.

### Additional information

No additional information is available for this paper.
